# Molecular Biomarker Analyses Using Circulating Tumor Cells

**DOI:** 10.1371/journal.pone.0012517

**Published:** 2010-09-08

**Authors:** Elizabeth A. Punnoose, Siminder K. Atwal, Jill M. Spoerke, Heidi Savage, Ajay Pandita, Ru-Fang Yeh, Andrea Pirzkall, Bernard M. Fine, Lukas C. Amler, Daniel S. Chen, Mark R. Lackner

**Affiliations:** 1 Department of Oncology Biomarker Development, Genentech, Inc., South San Francisco, California, United States of America; 2 Department of Molecular Diagnostics and Cancer Cell Biology, Genentech, Inc., South San Francisco, California, United States of America; 3 Department of Biostatistics, Genentech, Inc., South San Francisco, California, United States of America; 4 Department of Oncology Exploratory Clinical Development, Genentech, Inc., South San Francisco, California, United States of America; University of Medicine and Dentistry of New Jersey, United States of America

## Abstract

**Background:**

Evaluation of cancer biomarkers from blood could significantly enable biomarker assessment by providing a relatively non-invasive source of representative tumor material. Circulating Tumor Cells (CTCs) isolated from blood of metastatic cancer patients hold significant promise in this regard.

**Methodology/Principal Findings:**

Using spiked tumor-cells we evaluated CTC capture on different CTC technology platforms, including CellSearch® and two biochip platforms, and used the isolated CTCs to develop and optimize assays for molecular characterization of CTCs. We report similar performance for the various platforms tested in capturing CTCs, and find that capture efficiency is dependent on the level of EpCAM expression. We demonstrate that captured CTCs are amenable to biomarker analyses such as HER2 status, qRT-PCR for breast cancer subtype markers, KRAS mutation detection, and EGFR staining by immunofluorescence (IF). We quantify cell surface expression of EGFR in metastatic lung cancer patient samples. In addition, we determined HER2 status by IF and FISH in CTCs from metastatic breast cancer patients. In the majority of patients (89%) we found concordance with HER2 status from patient tumor tissue, though in a subset of patients (11%), HER2 status in CTCs differed from that observed in the primary tumor. Surprisingly, we found CTC counts to be higher in ER+ patients in comparison to HER2+ and triple negative patients, which could be explained by low EpCAM expression and a more mesenchymal phenotype of tumors belonging to the basal-like molecular subtype of breast cancer.

**Conclusions/Significance:**

Our data suggests that molecular characterization from captured CTCs is possible and can potentially provide real-time information on biomarker status. In this regard, CTCs hold significant promise as a source of tumor material to facilitate clinical biomarker evaluation. However, limitations exist from a purely EpCAM based capture system and addition of antibodies to mesenchymal markers could further improve CTC capture efficiency to enable routine biomarker analysis from CTCs.

## Introduction

Oncology drug discovery efforts are increasingly focused on targeted therapies that inhibit major nodes of oncogenic signaling pathways. A key to successful development of such agents is the ability to pre-select patients that will experience clinical benefit through molecular analysis of tumor tissue and the identification of predictive biomarkers that can match a drug with appropriate patients [1], [2], [3], [4]. Examples that illustrate the power of this approach are the HER2-targeting antibody trastuzumab (Herceptin®), which was successfully developed specifically in patients with tumor overexpression or amplification of HER2 [5], and the EGFR tyrosine kinase inhibitors gefitinib (Iressa) and erlotinib (Tarceva®), which have shown dramatic anti-tumor activity in patients whose tumors harbor oncogenic mutations in EGFR [6].

Available and representative tumor tissue is essential for biomarker assessment but can be difficult to obtain from patients with certain tumor types. An example is advanced stage non small-cell lung cancer (NSCLC), where surgery is frequently not a component of treatment and diagnosis is done with small biopsies or fine needle aspirates that yield only very limited tissue quantities [7]. Even in cases where primary tissue is available, the samples may not be representative of a patient's metastatic disease, which may arise many years after diagnosis and after a variety of therapeutic interventions. A primary example is prostate cancer, which often presents with multifocal localized disease but can often have a long indolent period of 10–15 years before it progresses to advanced, hormone resistant metastatic disease [8]. Furthermore, obtaining tissue from a fresh biopsy is challenging in this indication as the metastatic lesions are often localized to bone [9], [10]. Even if such material can be obtained, it is unclear whether a biopsy from a single site is representative of the majority of metastatic lesions and cases of marked heterogeneity have been reported [10]. Similar considerations are also true for metastatic breast cancer, where tumor samples may be limited to tissue from the primary disease site, which again, may be separated from disease recurrence by both time and treatment [11]. Collection of representative tumor material is clearly an important hurdle that must be overcome in successful biomarker development.

It has been known for over a hundred years that disseminated tumor cells can be found in the circulation of patients with metastatic cancer [12], and it has been hypothesized that these circulating tumor cells (CTCs) may represent cancer stem cells or a high metastatic potential cellular population [13]. In recent years, significant effort has been put into developing technologies that achieve specific and sensitive detection and capture of CTCs [14], [15], which is a major challenge since as few as one CTC may be found in the background of 105–106 peripheral blood mononuclear cells [16]. The CellSearch® platform uses immunomagnetic beads coated with antibodies to Epithelial Cell Adhesion Molecule (EpCAM) [17] to enrich for EPCAM-expressing epithelial cells, followed by immunostaining to confirm the presence of cytokeratin staining and absence of the leukocyte marker CD45 to confirm that captured cells are epithelial tumor cells [18]. The number of cells captured in this assay has been prospectively demonstrated to have prognostic significance for breast, colorectal and prostate cancer patients with advanced disease [19], [20], [21], [22].

In addition to prognostic utility, CTCs are an attractive alternative to tumor tissue for biomarker analysis that might help address some of the challenges described above [2], [13], [23]. One reason is accessibility and ease of collection, since CTCs can be obtained from a routine blood draw with minimal risk and inconvenience to the patient compared to a fresh biopsy. Another appealing facet of CTCs as a surrogate diagnostic tissue is the idea that CTCs could constitute a “liquid biopsy” and provide real-time information about the patient's current disease state [24], [25]. Analysis of biomarker status in CTCs collected prior to treatment could potentially be used to select an appropriate targeted therapy, while repeated longitudinal sampling during treatment could be used to detect appearance of resistance markers and potentially enable switching to a more appropriate therapy. For CTCs to fill this important niche, it is essential to demonstrate that they share molecular characteristics with a patient's solid tumor masses and that biomarker status in CTCs is reflective of biomarker status in neoplastic cells within tumor masses. An important recent advance that may facilitate molecular characterization of CTCs is the CTC-Chip, a microfluidic based CTC capture device where blood flows through a chamber containing thousands of microposts coated with anti-EpCAM antibodies to which the CTCs bind [26]. Importantly, reports on the CTC-Chip claim a significant increase in CTC counts and purity in comparison to the CellSearch® system [26], [27]. Both platforms have shown some prior evidence of utility for downstream molecular analysis. Examples include immunofluorescence for IGF-1R and the DNA damage response marker, gH2AX, in Phase I studies [28], [29], EGFR[7] and HER2[30] status in breast cancer, FISH for PTEN and FISH and RNA for TMPRSS2-ERG fusion in prostate cancer[24], [31], and genotyping for EGFR mutations in lung cancer[27].

Here we report a series of experiments that address two important challenges that must be answered if CTCs are to be routinely used for biomarker assessment. First, we compared the technical feasibility of isolating CTCs on different platforms, including CellSearch® and two commercially available CTC-chip platforms, and then used the captured CTCs for various downstream molecular analyses commonly used in biomarker assessment. Second, we evaluated whether the status of biomarkers such as HER2 in captured CTCs faithfully reflects biomarker status in matched tumor samples. To our knowledge, this is the first head-to-head comparison of CellSearch® and CTC-chip technologies, and overall we found similar performance in terms of CTC enumeration and the influence of EpCAM expression levels on capture rates. Secondly, our findings indicate that captured CTCs are amenable to biomarker analyses such as HER2 status, qRT-PCR for breast cancer subtype markers, KRAS mutation detection, and EGFR staining by IF. Finally, we find that the status of biomarkers such as HER2 in captured CTC generally reflects biomarker status in matched tumor samples.

## Materials and Methods

### Ethics Statement

#### Breast cancer patient samples

Patient blood was procured for this study by Open Biosystems (www.openbiosystems.com). All specimens were obtained with written informed consent and collected using a protocol approved by the Western Institutional Review Board (www.wirb.com). Patient information: All patients were Stage IV metastatic breast cancer patients, currently on active treatment. A list of patient information including HER2 testing and available treatment history is provided in Table S1.

#### Non-small-cell lung cancer patient samples

All patient samples were obtained as part of an ongoing Phase II clinical trial and is listed at http://clinicaltrials.gov/ct2/show/NCT00855894. All samples were collected and analyzed with written informed consent.

### EpCAM Expression Analysis

Expression of EpCAM and other genes in a panel of breast cancer cell lines was determined by microarray profiling on Affymetrix HGU133Plus_2.0 chips (Santa Clara, CA), as previously described [32], [33]. To examine the expression of marker genes in primary tumors, gene expression profiles were extracted from the commercially available database BioExpress (GeneLogic, Gaithersburg MD). Cell surface expression of EpCAM in breast cancer cell lines was evaluated by FACS (BD Caliber) using an anti-EpCAM biotinylated antibody (R&D Systems, BAF960) and streptavidin secondary.

### Tumor-cell Spike-in into Blood

Ten milliliters (ml) of blood from healthy donors were collected in appropriate blood tubes: CellSave for CellSearch, ACD for OncoCEE and EDTA for On-Q-ity CTC-chips. Tumor cells from culture cell lines were made into a 5000 cell/ml suspension and necessary volumes were spiked into the 10 ml blood tube of normal donor blood. Concurrently, an additional five spike-in volumes were dispensed onto microscope slides for manual counting to determine mean and range for spike-in. CTC counts obtained from CellSearch® or CTC-Chip platforms were divided by mean CTC count (based on spike-in count calculation) to determine percent CTC recovery, for additional detail see Figure S3. *Time delay analysis*: Spiked blood samples were stored at ambient temperature and analyzed in house at Genentech at the indicated time or sent to the collaborators and simultaneously analyzed on their platforms at 24 h and 48 h time points.

### CTC Technologies and CTC Enumeration

Samples run on CellSearch® were evaluated either at Genentech or at one of two reference laboratories as indicated in the figure legend. Reference Lab 1 is Apocell Biosciences (Houston, TX) and Reference Lab 2 is Veridex LLC Pharma Services (Huntington Valley, PA). CTC enumeration on CellSearch® was carried out according to manufacturers training and protocol [34], Veridex LLC (Raritan, NJ). CTC enumeration and analysis on the microfluidic CTC-chips were run at two reference labs that manufacture their own CTC-chip platforms: OncoCEE microchannel (Biocept Inc, San Diego, CA) and On-Q-ity's CTC-Chip (On-Q-ity, Boston MA and previously CELLective Dx, Menlo Park, CA). Cells were scored as CTCs on both CTC-chip platforms as per the following criteria: Cytokeratin+, CD45- and DAPI+. All samples sent for analyses on the CTC-chip platforms or at reference labs were blinded to them.

### EGFR and HER2 Immunophenotyping Assays

Spiked blood samples were processed on CellSearch® using the CellSearch® Tumor Phenotyping Reagent EGFR or HER2 (Veridex, LLC). H-scores were calculated using the method of McCarty, *et al* [35].

### qRT-PCR Molecular Subtyping Assay

Breast cancer cell lines corresponding to the breast molecular subtypes: Basal-like (HCC70), Luminal (T47D) and HER2 amplified (SKBR3) cells were spiked into normal donor blood collected into EDTA tubes, using donor blood alone as a control. Samples were processed on CellSearch® using the CellSearch RUO Profile kit (Veridex LLC). RNA was extracted from the isolated CTCs and qRT-PCR analysis performed using a TaqMan® low density array, TLDA, (Applied Biosystems, Foster City CA) with subtype specific genes [36].

### HER2 FISH Assay

FISH analysis on CellSearch® was done by Veridex Clinical Research Solutions (Veridex, Huntington Valley, PA) using the RF Poseidon™ Rare Cell HER-2 FISH assay (Veridex LLC). CTCs were captured and enumerated in the cartridge using the CellSearch® Assay. For FISH processing CTCs were fixed in the CellSearch® cartridge and hybridized with the HER-2/SE17 FISH assay. CTCs were relocated and scored using a CellTracks® Analyzer modified with a 40x objective and special software for relocating CTCs. FISH analysis on the OncoCEE Microchannel CTC-Chip was done by Biocept, Inc, using the PathVysion HER2 FISH kit (Abbott Laboratories, Abbott Park, IL). CTCs were first identified by standard criteria (CK+, CD45-, DAPI+). In addition, an automated cell locator was used to record the X and Y coordinates of each CTC. Following FISH processing, CTCs were re-identified based on location and cytokeratin positive staining and nuclei were scored for copies of HER2 and CEP17. HER2 gene amplification was defined as a ratio of >2 for hybridization signals from a HER2 locus specific probe to Chromosome 17 probe, and an average number of gene copies/cell of 4 or greater.

### KRAS Mutation Detection Assay

KRAS mutant H2122 cells were spiked into blood (10, 100, 1000 or 10000 cells in 10 ml blood). Spiked-in tumor cells were captured on CellSearch® using the RUO Profile kit. CTCs bound to ferrofluids were separated using the Magcellect magnet (R&D systems, Minneapolis, MN) and DNA extracted using the Picopure DNA extraction kit (Molecular Devices, Sunnyvale, CA) by overnight lysis. The full volume of extracted DNA was subjected to a preamplification reaction using KRAS exon 2 primers followed by PCR on the Fluidigm Digital Array platform (Fluidigm, South San Francisco, CA) using a Taqman genotyping assay for the KRAS mutation G12C, with the probe designed to detect the presence of the mutant amplicon. Primer and probe sequences were as follows: reverse primer; GCTGTATCGTCAAGGCACTCTTG, forward; GGCCTGCTGAAAATGACTGAA and mutant MGB VIC labeled probe; TTGGAGCTTGTGGCG.

## Results

### Prevalence of EpCAM expression and impact of differences on CTC capture

**Figure 1:**
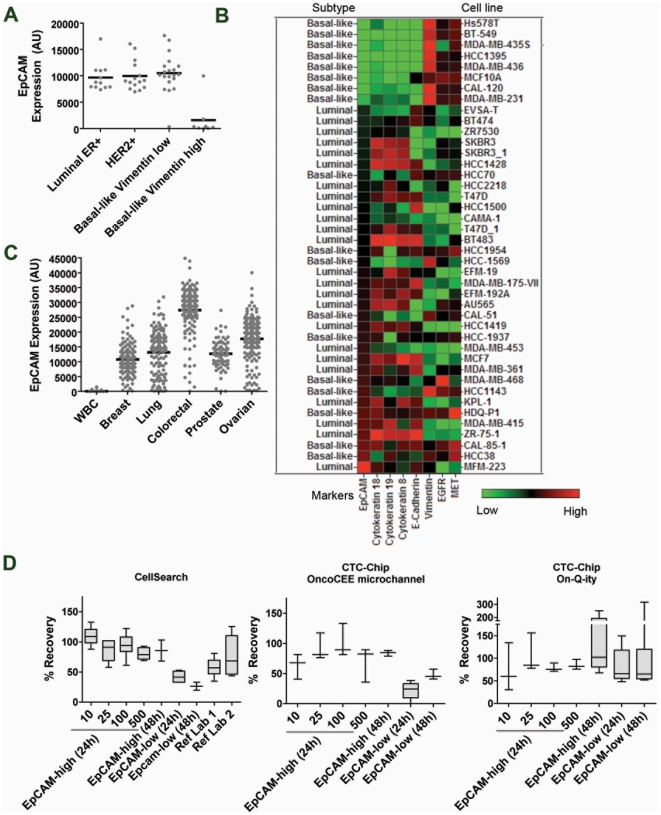
Evaluation of EpCAM as a marker for capturing CTCs. A. EpCAM expression in breast cancer cell lines grouped by molecular subtype. B. Expression of EpCAM in relation to other epithelial and mesenchymal markers in breast cancer cell lines. C. EpCAM expression in different tumor types and in white blood cells (WBC). D. Spike-in CTC recovery on CellSearch® (left panel) in EpCAM high and EpCAM low cells when analyzed at 24 h or 48 h post spike-in at Genentech or two reference labs (Ref Lab). In the EpCAM-high (24 h) group, samples are graphed by individual spike-in cell count (10–500 cells) and pooled together for all other groups.

EpCAM (also know as TACSTD1) is well characterized as a marker of epithelial tumor cells [37] and anti-EpCAM antibodies have been widely used in the capture of CTCs [14]. As a first step in evaluating CellSearch® in comparison with the CTC-Chip platforms, all of which use EpCAM expression as the basis for CTC capture, we looked at the utility of EpCAM as a universal marker expressed on cancer cells by evaluating its expression in cell lines and across diverse tumor types. We determined the distribution of EpCAM mRNA expression across 50 breast cancer cell lines representive of luminal, HER2 amplified and basal-like breast cancers (Figure 1A) [32], [36]. EpCAM expression was high, >7000 Affymetric units (AU), across the majority of cell lines (Figure 1a). However, about 20% of the cell lines showed little to no EpCAM expression (<500 AU). Interestingly, cell lines with low EpCAM expression were primarily from a subset of the basal-like subtype that showed high expression of the mesenchymal marker vimentin and low expression of epithelial markers such as cytokeratins 8, 18 and 19 and E-cadherin (Figure 1B). These cell lines have previously been described as the Basal B subgroup and known to have higher expression of mesenchymal markers, as well as certain stem cell-like properties and a more invasive phenotype [36]. Importantly, *EGFR* and *c-Met* were highly expressed in this subset, suggesting that antibodies against these cell surface markers could have utility in capturing this subset of breast cancer cells. This data suggests that CTCs may be difficult to capture using a purely EpCAM-based capture mechanism in a subset of basal-like breast cancers.

We also evaluated the distribution of EpCAM at the mRNA level in patient tumor samples and found that while the majority of tumor types showed high EpCAM expression, we again noticed that a subset of samples from each tumor type had lower EpCAM expression (Figure 1C). In particular, lung and ovarian cancer samples had a greater percentage of tumor cells with very low expression (Figure 1C). Again, we observed that low EpCAM expression was associated with high vimentin expression (Figure S1), suggesting that capture of mesenchymal tumors may be challenging with EpCAM based-methods and that other capture antibodies may be required to capture the full range of CTCs.

### Evaluation of EpCAM-based capture using commercially available CTC technologies

We first investigated a number of variables that could conceivably affect CTC recovery and enumeration using the CellSearch® platform, including EpCAM expression, CTC number, time to analysis, and variations when samples are analyzed in different reference labs using the same platform. For this purpose we chose two breast cancer cell lines: SKBR3, which has high EpCAM mRNA expression (>7000 AU) and CAL-120, which has low mRNA expression (<500 AU). Expression at the protein level was confirmed by FACS analysis (Figure S2). We then developed a tumor cell spike-in protocol that enabled us to reproducibly spike-in cultured tumor cells into normal blood and recover as few as 10 spiked-in tumor cells from 7.5 ml whole blood with a typical variability of +/−30% (Figure S3). We next spiked tumor cells from SKBR3 and CAL-120 into whole blood and tested the recovery rate under different conditions using the CellSearch® and CTC-chip platforms.

On the CellSearch® platform, we found that average CTC recovery in the high EpCAM SKBR3 cell line was 75% or better (Figure 1D), whereas in low-EpCAM CAL-120 cells CTC recovery was significantly lower with a mean of 42% (p = 2.0 E-4, Student's t-Test). We found no significant change in recovery between 24 and 48 hours post analysis, but we observed that the EpCAM-low cells did show a trend toward decreased recovery at 48 hours. We also shipped SKBR3-spiked samples to two reference labs for analysis to evaluate the effects of shipping, transport and multiple operators on CTC recovery. Average CTC recovery using CellSearch® was lower at a mean of 58% in the case of Reference Lab 1 (p = 1.0 E-6) in comparison to analysis done at Genentech. In the case of Reference Lab 2, the data appeared more variable but overall recovery was not statistically different (p = 0.25).

We next evaluated two different commercially available microfluidic CTC-chip platforms and compared the results to CellSearch®. In general, average recovery of high EpCAM expressing SKBR3 cells on both CTC-chip platforms was >75% and was comparable to CellSearch® except at the 10-cell spike in level which was less robust and varied to below 50% recovery on both CTC-Chip platforms (Figure 1D, middle and right panels). In EpCAM-low CAL-120 cells, average CTC recovery was below 50% on CellSearch® and similarly low on the OncoCEE microchannel chip platform (p = 0.42), but improved to >50% on the On-Q-ity CTC-chip (p = 0.001). One caveat to the higher numbers observed on this platform is that CTC recovery was substantially above 100%. Spike-in recovery up to 130% could be attributed to the +/−30% error rate associated with spike-in, but beyond that might indicate non-specificity in the assay, with blood cells possibly erroneously scored as CTCs.

### Technical feasibility of molecular biomarker assays using CTCs

We next tested technical feasibility of using isolated CTCs in assays that are most commonly used for biomarker assessment: protein expression by immunofluorescence, DNA amplifications by FISH, mRNA transcript expression by qRT-PCR and oncogenic mutations in DNA by a q-PCR genotyping assay.

**Figure 2:**
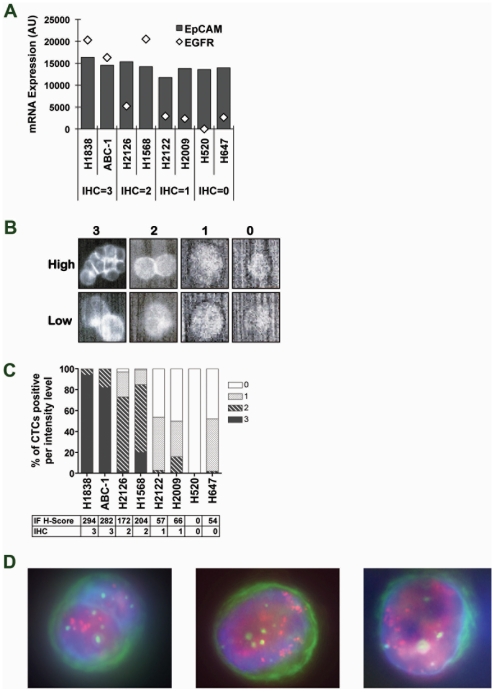
EGFR IF and HER2 FISH in CTCs. A. mRNA expression of EGFR (diamonds) or EpCAM (bars) in NSCLC cell lines. IHC scores for EGFR from tissue microarrays are indicated below. B. EGFR immunofluorescence (IF) scoring criteria for CTCs. For each scoring level, the range of high and low expression are shown. C. EGFR IF scoring of spiked tumor cells isolated from blood. The weighted H-score from CTC analysis and corresponding IHC score for that cell line is listed below for each sample. D. HER2 FISH assay in captured SKBR3 cells on the OncoCEE microchannel platform. Cells are stained with anti-cytokeratin antibody (green), DAPI (blue), FISH probes against HER2 (red dots) and a centromeric probe, CEP17 (green dots).

We tested if EGFR expression could be accurately determined by immunofluorescence (IF) in CTCs using eight non-small-cell lung cancer (NSCLC) cell lines with varying levels of EGFR mRNA and known protein expression levels from IHC analysis on tissue microarrays (Figure 2A). These cell lines were chosen such that they had similar high EpCAM expression to ensure capture was not a variable in this analysis. Using an EGFR antibody, we evaluated the range of expression seen in CTCs isolated from tumor-cell spike-in blood samples from four of the eight cell lines. Instead of scoring EGFR by presence or absence of staining as previously described[38], we developed a semi-quantitative scoring criteria for EGFR expression levels based on staining intensity and membrane localization (Figure 2B). These scoring criteria were then used to score EGFR levels in captured spike-in CTCs from the remaining four cell lines (Figure 2C). We observed generally excellent agreement between EGFR staining in CTCs and EGFR levels determined by IHC on cell pellets. Interestingly, not all CTCs scored identically within a sample and in fact the analysis indicated some heterogeneity in EGFR expression. A weighted H-Score was then computed using the IF score and the percentage of cells with that score, to provide a single value for EGFR expression. H-score values correlated well with the EGFR mRNA expression level in the cell lines (R2 = 0.91) and the cell surface expression of these cell lines by FACS analysis (Figure S4), indicating biomarker analysis based on levels of protein determined by IF may be feasible in captured CTCs.

HER2 gene amplification status by fluorescence in situ hybridization (FISH) assay is routinely used to determine patient eligibility for anti-HER2 therapies such as trastuzumab and lapatinib [39], [40], [41]. We sought to determine whether a HER2 FISH assay could be reliably used to detect HER2 amplification in CTCs. Tumor cells from the HER2 amplified cell line SKBR3 were spiked into whole blood and cells were captured on the OncoCEE microchannel chip platform and analyzed for HER2 FISH (Figure 2D). We tested the robustness of this assay by evaluating FISH on samples with as few as 2 CTCs and many as 159 CTCs (Table 1). All samples with 3 or more CTCs showed a HER2 to centromere 17 ratio of greater than two and would thus be classified as HER2-amplified according to standard criteria [42].

**Table 1:** Performance of the HER2 FISH assay on the OncoCEE platform.

Sample	CTC Count	CTCs FISHed	HER2/CEP17 ratio
1	159	20	2.949
2	151	20	3.13
3	140	20	2.74
4	72	20	3.74
5	41	20	3.688
6	37	20	3.33
7	17	16	failed
8	10	10	2.86
9	10	10	3.04
10	3	3	3.33
11	5	2	4.22
12	2	2	1.88

Spike-in CTC count upon capture and post-FISH processing and HER2/CEP17 ratios are indicated across a range of tumor cell spike-ins analyzed on the OncoCEE microchannel platform.

**Figure 3:**
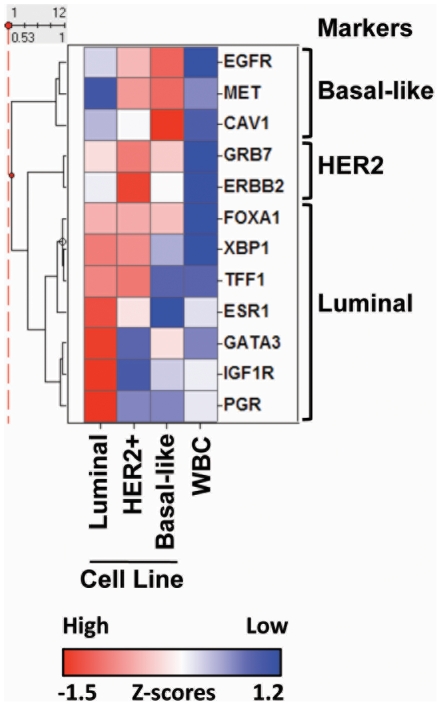
qRT-PCR assay for molecular subtyping of breast cancer in CTCs. A. 100 cells were spiked into normal donor blood from a luminal (T47D), HER2+ (SKBR3) and a basal-like breast cancer cell line (HCC70) or negative control (WBC) and were isolated using the CellSearch® platform, followed by qRT-PCR analysis with a panel of genes specific for the three corresponding breast cancer subtypes. Heatmap shows hierarchically clustered z-score normalized Ct values for each gene.

We also evaluated whether qRT-PCR based multi-gene expression analysis can be done from CTCs isolated on the CellSearch® platform using blood samples spiked with breast cancer cell lines representing each of the major breast cancer subtypes. Specifically, we used T47D ER+ luminal cells, SKBR3 HER2-amplified cells, and HCC70 basal-like cells [32], [33]. RNA from captured spike-in CTCs was subjected to qRT-PCR analysis using a panel of genes derived from the Sorlie “intrinsic set” of genes that can distinguish between the molecular subtypes based on subtype specific expression patterns [43], including estrogen receptor (*ER*) and progesterone receptor (*PGR*) for the luminal subtype, *HER2* and *GRB7* from the *HER2* amplicon, and *EGFR* and *c-Met* from the basal-like subtype. Gene expression analysis on this panel of 12 genes show the expected subtype-specific gene expression pattern for each of the cell lines at both the 100 cell and 10 cell spike-in range (Figure 3 and Figure S5, respectively), but did not clearly discriminate between subtypes when less than 10 cells were spiked in. The sensitivity limitation of this assay is affected by the leukocyte background, which is in the range of 1000–3000 cells per sample dependent on donor, and would give CTC:WBC ratios of ∼1∶100 for sample with 10 CTCs and ∼1∶10 for sample with 100 CTCs.

**Figure 4:**
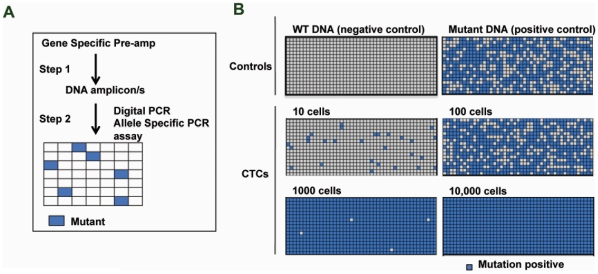
KRAS mutation detection assay in CTCs. A. Schematic of digital PCR based mutation detection assay. B. Results of the KRAS G12C mutation assay on Digital PCR arrays starting from DNA isolated from KRAS mutant tumor H2122 cells spiked into whole blood in the indicated numbers.

Another potentially important use of CTCs for biomarker analysis is detection of oncogenic mutations that may predict response to targeted agents [1]. Genotyping assays such as allele-specific PCR often only have sensitivity down to approximately 1% mutant DNA in a background of wild-type DNA [44], so wild-type copies of the gene of interest from contaminating WBCs co-isolated with CTCs might hamper detection of CTC-specific mutations. We developed a mutation detection assay that could tolerate contamination by wild-type DNA by starting with a gene specific amplification on total CTC lysate, followed by Taqman genotyping assays using digital PCR arrays, a critical step to increase the specific concentration of the mutant DNA relative to WT DNA (Figure 4A). Assay performance was demonstrated using KRAS mutant H2122 cells spiked into blood at levels ranging from 10 cells up to 10,000 cells per 7.5 ml of blood (Figure 4B). The KRAS G12C mutation was detectable in DNA from as few as 10 captured cells, with increasing numbers of positive wells observed with the higher cell numbers.

### Evaluation of EGFR expression and HER2 status in CTCs from patients with metastatic disease

**Figure 5:**
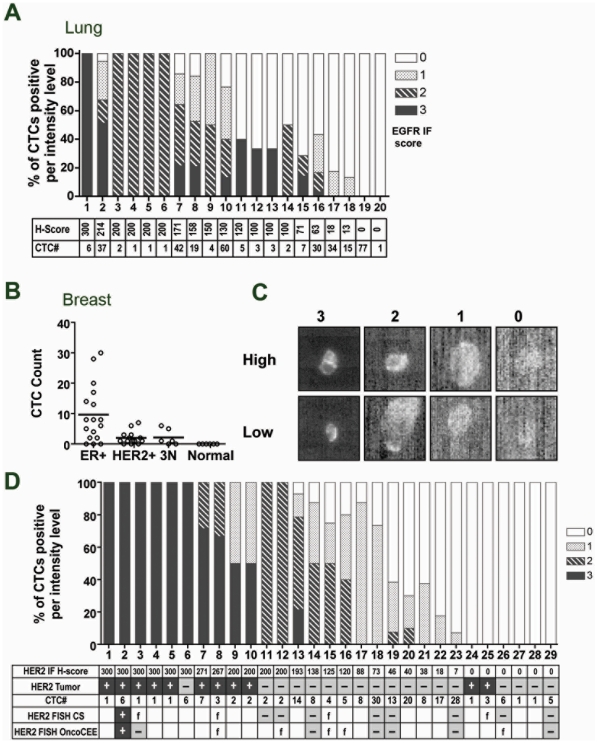
EGFR expression and HER2 status in CTCs from metastatic cancer patients. A. EGFR scoring in CTCs from metastatic lung cancer patient samples, scored according to criteria described in Figure 2B. B. CTC counts from 39 metastatic breast cancer patient samples listed by subtype. C. HER2 IF scoring criteria showing the high and low range of expression. D. Quantification of HER2 scoring in CTCs. H-score for HER2 IF in CTCs, HER2 status in patient tumor, CTC counts and HER2 FISH results in CTCs are listed in table below. HER2 FISH was performed on CellSearch (CS) or on the OncoCEE microchannel (OncoCEE). For HER2 FISH, (+) indicates a positive FISH result, (-) indicates a negative result and (f) indicates that the FISH failed because no CTCs were detected.

We used the EGFR IF assay to evaluate EGFR expression on samples from 34 patients with metastatic NSCLC enrolled in a phase II clinical trial3. Out of the 34 patients who had blood collected for CTC analysis prior to starting therapy, 24 patients (70%) had at least one evaluable CTC with 50% of patients having 3 or more CTCs (Figure 5A), which is somewhat surprising given the generally low level of CTCs reported in NSCLC patients [18]. In the 20 patients that EGFR expression data was collected, we observed a range of EGFR expression and a mixture of homogenous and heterogenous EGFR staining between samples. A few patients had very heterogeneous expression with CTCs that spanned from 0–3 in staining intensity and this was best observed in patients with higher CTC counts (>7 CTCs), though some patients with high CTC counts also had homogeneous EGFR staining. These studies suggest it may be feasible to quantitate cell surface expression of candidate biomarkers on CTCs in patients with metastatic NSCLC, however, only about 50% of patients had sufficient CTCs (>3) for assessing heterogeneity in expression, suggesting that improvements in capture technologies will be required to fully enable biomarker studies in CTCs from NSCLC patients.

We next evaluated HER2 status in CTCs from 38 patients with advanced metastatic breast cancer. CTC counts in the patient samples indicated a distinct distribution in different subtypes. ER+ patients had the highest CTC counts (range: 0–30, median:8), while lower CTC counts were seen in HER2+ patients (range: 0–7, median:1) and in triple negative patients (range: 0–6, median:1) (Figure 5B). To determine if the difference in CTC counts between subtypes was significant, we used non-parametric statistical methods. A population based Poisson distribution assumption would not be a good fit here because the variance in CTC counts is higher than expected from such a model. There was a significant difference in CTC counts found among all subtypes (Kruskal-Wallis method p-value  = 0.004) or between ER+ and others (Mann-Whitney p-value  = 0.02).

Twenty-nine of these patients (76%) had at least 1 CTC that was evaluable using the CellSearch® platform. We evaluated HER2 at the protein (IF assay) and DNA levels (FISH assay) and compared HER2 status in CTCs to HER2 status in patient tumor samples determined by tumor IHC/FISH on archival tissue. A previous report evaluating HER2 expression in CTCs on the CellSearch® platform scored HER2 positivity based simply on the presence of HER2 staining in 50% of the CTCs and reported discordance in HER2 status when compared to primary tumor in a third of the patient samples [30]. To allow more direct comparisons to the HER2 IHC scores obtained from matched tumors samples, we developed a scoring system to quantitate HER2 expression using a 0–3+ score in CTCs (Figure 5C) and then computed an H-score to provide a weighted score based on the number of CTCs with a given level of expression.

The HER2 IF staining and computed H-score for the 29 patients with at least one evaluable CTC is shown in Figure 5D, and we compared these scores with tumor IHC/FISH results. Nine out of 12 patients (75%) whose CTCs had an H-score of 200 or more were HER2+ based on IHC/FISH analysis of archival tissue. Correspondingly, 15 out of 17 patients whose CTCs had an H-score of less than 200 had tumor IHC scores of 0, 1+ or 2+ and were HER2- based on IHC/FISH analysis of archival tissue. Thus, we observed 89% concordance overall (24 of 29 patients) between HER2 status in CTCs and HER2 status in neoplastic tissue samples. Under the assumption that a patient's HER2 status in the tumor represents the truth, we calculated what number of CTCs would best balance the false positives (Type I error) and false negatives (Type II error) and found that >3 CTCs can yield robust results for determination of HER2 status (Table S2 and Figure S6). We also asked if HER2 status changed with time or treatment. In replicate sampling in a small subset of the same patients, we found that HER2 status was generally stable in samples from the same patient (Figure S7). Furthermore, a subset of the HER2 positive patients in this study were on trastuzumab treatment at the time of blood collection, however, we found no significant difference in HER2 expression in the patient subset currently on treatment with trastuzumab (Figure S7).

We also performed HER2 FISH using both CellSearch® and the OncoCEE microchannel platform on CTCs in a subset of patients from the IF study where it was possible to obtain additional blood draws one to two months later. We found that nine out of 13 samples (69%) tested on the CellSearch® platform had one or more CTCs that could be evaluated by FISH (Figure 5D). HER2 status by FISH agreed with the HER2 status from archival tissue for all nine of these patients. Though only one of the four patients with HER2+ status in archival tissue had CTCs amenable for FISH in this analysis, HER2 amplification was detected in the majority of CTCs from this patient except in 3 out of 22 CTCs scored that had <4 copies of HER2, None of the 22 WBC scored had >2 copies of HER2. Overall, the FISH results were also concordant with the IF results for all of these patients. In comparison, six out of twelve samples (50%) tested on the OncoCEE microchannel platform yielded CTCs that could be scored in the HER2 FISH assay (Figure 5D). FISH results for five out of the six agreed with HER2 status from archival tissue and IF on CTCs, with the exception of patient 3, who was HER2+ based on archival tissue and CTC IF assay but FISH negative based on HER2 FISH analysis on the OncoCEE platform.

## Discussion

The advent of targeted cancer therapeutics has resulted in a paradigm shift from treating all patients with high dose chemotherapy to more personalized approaches based on tumor genetics and biology [1], [3], [4]. Targeted therapies have the potential benefit of greater efficacy and reduced toxicity, but also require predictive biomarker assays to identify appropriate patients. A practical challenge to biomarker-based patient selection is the availability of relevant tumor material. Obtaining a tumor biopsy in a patient with advanced disease may be scientifically desirable but often is not a practical option. Circulating tumor cells thus have tremendous potential to change our approach to biomarker evaluation by providing a source of tumor material that is easily accessible through a blood draw. An additional potential benefit is that CTC molecular characteristics may in some cases be more representative of the patient's current disease than archival tumor tissue obtained years before at the time of diagnosis.

An important challenge that must be met if CTCs are to become widely used as a surrogate tissue for biomarker analyses is that they must be present in sufficient numbers to allow molecular characterization in the majority of patients. In the original report published on the CTC-chip, the authors reported an average CTC count of >50 CTC/ml and that 100% of patients across multiple indications showed >5 CTCs/mL of blood [26], a significantly higher prevalence and number than are typically described for the CellSearch® platform [18]. Higher CTC counts on the CTC-chip platform have been attributed to the unique engineering and microfluidic properties of the device, since careful theoretical modeling was used to optimize the balance between velocity and sheer forces and thus maximize capture of CTCs on microposts [26]. Gentle laminar flow across CTC-chip microposts coated or “functionalized” with anti-EpCAM antibodies has been suggested to result in higher yields and purity of CTCs by maximizing CTC contact with capture antibodies and minimizing forces that could cause disruption of cellular integrity [45]. These studies suggested that the CTC-chip technology might prove superior to the CellSearch® platform in terms of utility for biomarker analyses based on substantially increased sensitivity and greater yields of CTCs for downstream molecular analyses [45]. However, a side-by-side comparison of CTC-chip platforms with CellSearch® has not been previously reported. In this study, we compared the performance of commercially available CTC-chip devices to the CellSearch® platform using the same conditions and samples from our cell line spike-in model system, as well as samples from patients with advanced metastatic breast cancer. We found that both CellSearch® and the two CTC-chip platforms tested here were effective at capturing high-EpCAM expressing cells, but efficiency fell to below 50% for cells expressing lower EpCAM levels. The On-Q-ity CTC-chip platform showed slightly improved capture in this context, but recoveries greater than 100% suggested some non-specificity in this assay. Thus, both CellSearch® and the CTC-chip platforms may have difficulty capturing CTCs from more mesenchymal tumor types, and could benefit from additional capture antibodies directed at antigens with more prevalent expression in mesenchymal tumors. Indeed, some evidence points to CTCs undergoing epithelial mesenchymal transition (EMT) as part of the process of dissociating from the original tumor mass and initiating the metastatic process [46], [47], further suggesting that non-EpCAM-based capture methods could benefit both CellSearch and CTC-chip platforms. To this point, somewhat surprisingly, we found higher CTC counts in ER+ breast cancer patients than in HER2+ or triple negative patients. This may be a reflection of the underlying molecular subtype and gene expression patterns of these tumors since we observe high EpCAM expression in the luminal subtype of breast cancer and weak expression of EpCAM in the basal-like subtype which instead expresses mesenchymal markers such as vimentin, EGFR and MET. With regard to capturing CTCs from patient samples, CellSearch® and the OncoCEE microchannel platform performed similarly (69% and 50% prevalence of patients with CTCs, respectively), and in both cases the captured CTCs were amenable to downstream biomarker analysis by FISH assay. Thus, our overall results suggested relatively similar performance in terms of CTC capture between CellSearch® and the CTC-chip platforms in terms of both sensitivity and yield. There are several possible explanations as to why our findings differ from previous reports. First, some of our comparative studies were conducted using spiked-in cell lines, which could perform differently than CTCs from cancer patients on these platforms, since cell lines could conceivably be more robust than epithelial cells in peripheral circulation. Second, the CTC-chip platforms we evaluated may have diverged from the original platform in terms of materials used to construct the chips as well as the layout and architecture of the microposts and the rate of flow across the chip, possibly resulting in less efficient capture. Third, samples in the initial CTC-chip reports were collected and run at the same site and with no shipping step [26], [27], so it is possible that handling or delays in processing inherent to shipping may have compromised recovery of CTCs in our shipped samples compared to those studies. Further study will be required to understand the key variables that impact CTC recovery on the various platforms and determine whether performance is comparable under a variety of conditions, but a conclusion from our studies is that current commercially available biochip platforms are comparable to CellSearch® for capture and molecular characterization of CTCs.

Another challenge that must be met if CTCs are to truly have utility for biomarker applications is that captured cells must be amenable to commonly used biomarker assay formats such as IF, FISH, mutation detection, and qRT-PCR. In this report, we systematically evaluated the technical feasibility of using isolated CTCs for these common assay formats, with particular emphasis on the suitability of cells captured on the CellSearch® platform for these applications, since this is an FDA approved platform and now widely available in diagnostic labs. We found that captured CTCs were amenable to quantitative IF scoring for both EGFR and HER2 on the standard CellSearch® platform, and that the levels of spiked tumor cells isolated from blood were generally reflective of expression levels in the parent cell line. Furthermore, we found that even in a tumor type such as NSCLC, which has previously been reported to have a prevalence of only 20% of patients having any CTCs [18], we were able to capture at least one CTC and quantitate EGFR expression in blood samples from 24 out of 34 patients we evaluated. The higher prevalence of CTCs we observed may reflect the patient population enrolled in this clinical trial, since a recent paper reported that CTC counts in NSCLC patients increased with tumor progression and distant metastasis [48]. Although, evaluation of EGFR expression in CTCs has been described previously, the data in our report is notably different. Whereas Payne et al, looked simply at the presence or absence of EGFR expression, we developed a quantitative scoring criteria with four expression levels 0,1, 2 and 3 based on lung cancer cell lines that span the spectrum of EGFR expression observed in lung cancer from an IHC score of 0 to 3. Secondly, whereas Payne et al. looked at EGFR expression in breast cancer, we demonstrated the feasibility of this assay in lung cancer, where CTC characterization is thought to be significantly more difficult due to lower CTC counts. In addition, lung cancer poses a significant need for biomarker analysis from blood due to limited tissue availability. Taken together, our findings suggest that quantitative assessments of protein expression for drug targets such as EGFR and HER2 may be feasible in CTCs.

In addition, we found that nucleic acids prepared from CTCs captured using the CellSearch® RUO Profile kit were also amenable to biomarker assays including a qRT-PCR gene expression assay for breast cancer molecular subtype, and a PCR-based assay for KRAS mutations. These results suggest that in addition to its currently approved role for prognostic testing based on CTC enumeration, the CellSearch® platform may have utility in capturing CTCs that can be used for predictive biomarker analyses.

An important qualification to using CTCs for molecular biomarker detection and patient selection is that CTCs accurately represent the molecular characteristics of the tumor mass. HER2, in this regard, is a gold standard for biomarker validation because it is a well-characterized marker where the metrics for HER2 positivity have been tested and correlated with response to trastuzumab treatment [5], and FDA approved in vitro diagnostic kits exist for HER2 testing along with ASCO/CAP testing guidelines for appropriate testing [41]. We evaluated HER2 status by IF in CTCs from 29 patients with advanced metastatic breast cancer and known HER2 primary tumor status. A novel aspect of our study is that we focused on the feasibility of HER2 testing in CTCs from a heavily pretreated metastatic breast cancer patient population and comparison tested the two leading CTC platforms, CellSearch® and CTC-Chip. Although our patient population was heavily pretreated with chemotherapy, radiation therapy and trastuzumab, we nevertheless found 89% concordance between HER2 status in CTCs and HER2 status from archival tumor samples. Though the data showed generally good agreement between HER2 status in CTCs and archival tissue, the specific differences we observed may be due to several biological and/or technical factors and deserve some discussion. First, marked intratumoral heterogeneity for HER2 amplification has been well documented and is thought to represent subclonal diversity within tumor samples [49], so analysis of 1–5 CTCs might not be sufficient to detect HER2+ CTCs in a heterogeneous sample. To this point, we find that limiting analysis to samples with >3 CTCs significantly reduces the discordance with tumor HER2 status to just one patient using the HER2 IF assay for CTC characterization. Second, clinical testing for HER2 is known to result in both false positives and false negatives [41], so it is possible that some patients were misclassified based on the original IHC test. A false negative tumor IHC result could explain the situation in patient 6, in which all six CTCs uniformly stained 3+ but the tumor IHC result was HER2 negative. Similarly, patients 24 and 25 were both HER2+ by IHC but negative by CTC IF. Third, it is possible that HER2 status can change over the course of disease progression, such that HER2 amplification is acquired later, leading to a positive test result in CTCs in spite of a negative tumor IHC result. This could also explain the result in patient 6. Studies that have looked at HER2 status in matched primary and metastatic tumor samples have generally found concordance in the range of 90%, suggesting that HER2 status is generally stable [50], [51], [52], [53], [54], but some recent reports have shown lower concordance in the range of 60–70%, particularly when comparing between primary tumor and CTC HER2 status [25], [30], [55]. In comparison, our study reported a slightly higher concordance at 89% between primary tumor and CTCs. This difference may be attributable to the small sample sizes in all of these studies (under 75 patients), or the fact that HER2 status determined by immunofluorescence is not a standardized assay with common scoring criteria. For example, whereas we used an H-score cutoff of 200 for HER2 positivity, another report defined their cutoff as 50% of cells having HER2 positive staining[30]. An implication of all of these studies taken together is that HER2 amplification can apparently be acquired late in disease progression in some instances. Notably, several patients with HER2 negative primary disease but HER2 positive CTCs have been reported to show clinical benefit from trastuzumab treatment [25], possibly suggesting some diagnostic utility of HER2 characterization in CTCs for determining eligibility for HER2-targeting agents in patients with HER2 negative primary disease.

Overall, the data we provide in this study indicate that molecular characterization of CTCs may have utility in biomarker assessments in clinical trials, and that currently available platforms have utility in the isolation of CTCs that can be used for biomarker analyses. However, based on our assessment that >3 CTCs may be the minimum number of CTCs required to sample heterogeneity and minimize assay error, this criteria would only be met in about 50% of patients in both our lung and breast cancer studies and this is an important limitation of current technologies. In addition, based on our findings that EpCAM may be weakly expressed in certain subtypes of cancer, current technologies that limit capture to using EpCAM alone could significantly benefit from adding markers of mesenchymal phenotype to improve CTC counts and prevalence in patients. We anticipate that such improvements in CTC technologies along with future prospective predictive biomarker studies in CTCs from larger patient populations with temporally matched tumor tissue and treatment response data will conclusively establish a role for CTCs in molecular biomarker-based patient selection.

## Supporting Information

Figure S1 EpCAM expression is often lower in ovarian and lung tumor samples with high Vimentin expression. Affymetrix gene expression data for EpCAM from ovarian and lung tumors was binned by low or high Vimentin expression based on cutoff at 30th percentile. EpCAM expression was significantly lower in the vimentin high group using Student's t-Test in ovarian tumors (p = 0.0036) and lung tumors (p = 0.012).
(0.11 MB DOC)

Figure S2 EpCAM expression on the cell surface is correlated with mRNA expression a. EpCAM expression on the cell surface by FACS analysis. b. EpCAM expression on the cell surface in comparison to EpCAM mRNA expression in the same cell lines.
(0.05 MB DOC)

Figure S3 Schematic of spike-in protocol and spike-in statistics.
(0.26 MB DOC)

Figure S4 Correlating EGFR in CTCs with expression in parent cell line a. H-Score for EGFR expression in CTCs correlates well with EGFR mRNA expression in cell lines. b. H-Score for EGFR expression in CTCs correlates well with EGFR surface expression by FACS analysis in cell lines.
(0.09 MB DOC)

Figure S5 qRT-PCR assay for molecular subtyping of breast cancer from 10 CTCs. Tumor cells from a luminal (T47D), HER2+ (SKBR3)and a basal-like breast cancer cell line (HCC70) or negative control (WBC) were spiked into 10 ml donor blood and isolated using the CellSearch® platform. Cell lysates were subjected to multi-plex qRT-PCR analysis with a panel of genes specific for the three corresponding breast cancer subtypes. Heatmap shows hierarchically clustered z-score normalized Ct values for each gene.
(0.06 MB DOC)

Figure S6 Type I and Type II error calculated for HER2 IF CTC assay with increasing number of CTCs, using HER2 status in patient tumor as “truth”.
(0.03 MB DOC)

Figure S7 HER2 expression is largely unchanged in replicate sampling and on treatment with Herceptin. a. Quantitation of HER2 immunoflourescence (IF) by H-score in replicate samples from the same patients collected 1–2 months apart. b. HER2 IF H-score in HER2 positive patients who were either on Herceptin or alternate treatment (Other) at time of blood collection.
(0.05 MB DOC)

Table S1 Breast cancer patient and CTC characteristics. This table contains data for all 29 patients with evaluable CTCs. Hormone receptor status and HER2 test results is from patients pathology reports, unless unavailable in which case HER2 status was from information available from patient profile. Treatment at collection date was as available from patient profiles. CTC characteristics are as listed in Figure 6.
(0.81 MB DOC)

Table S2 Calculations for Type I and Type II error in the HER2 calls in the HER2 IF CTC assay with respect to HER2 status from patient tumor. (n =  number of patients, TP = true positive, FP = false positive, TN = true negative, FN = false negative).
(0.05 MB DOC)
