# Supplementary figures and images for: Molecular Biomarker Analyses Using Circulating Tumor Cells
Source: PLoS One. 2010 Sep 8;5(9):e12517. doi: 10.1371/journal.pone.0012517 (PMC2935889; doi:10.1371/journal.pone.0012517)

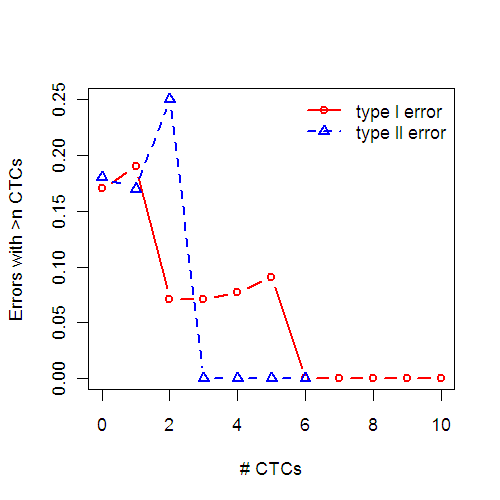

Supplement: Figure S6 — Type I and Type II error calculated for HER2 IF CTC assay with increasing number of CTCs, using HER2 status in patient tumor as “truth”. (0.03 MB DOC) [file pone.0012517.s006.doc]

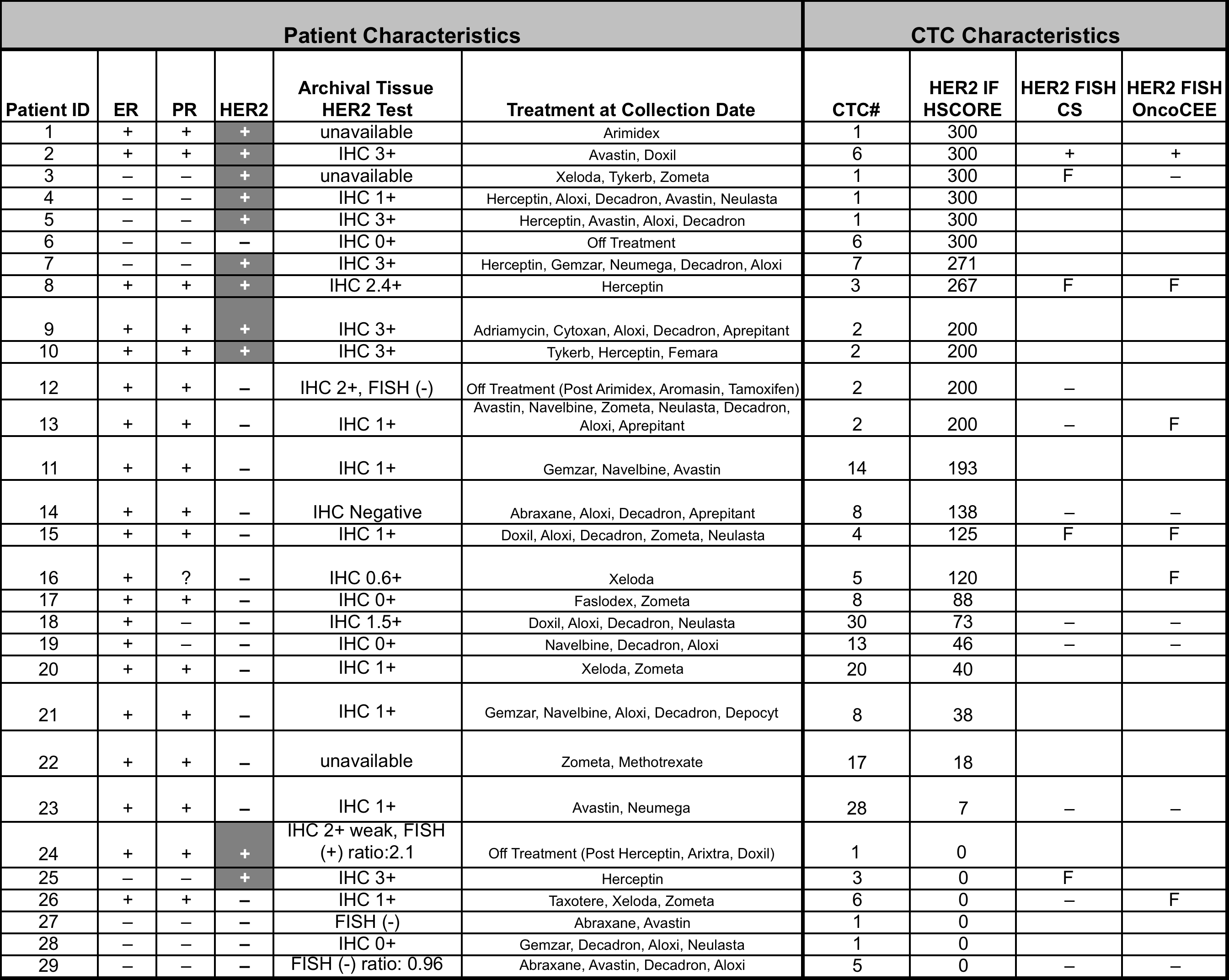

Supplement: Table S1 — Breast cancer patient and CTC characteristics. This table contains data for all 29 patients with evaluable CTCs. Hormone receptor status and HER2 test results is from patients pathology reports, unless unavailable in which case HER2 status was from information available from patient profile. Treatment at collection date was as available from patient profiles. CTC characteristics are as listed in Figure 6. (0.81 MB DOC) [file pone.0012517.s008.doc]
