# Supplementary material for: Molecular Biomarker Analyses Using Circulating Tumor Cells
Source: PLoS One. 2010 Sep 8;5(9):e12517. doi: 10.1371/journal.pone.0012517 (PMC2935889; doi:10.1371/journal.pone.0012517)
Supplement: Table S2 — Calculations for Type I and Type II error in the HER2 calls in the HER2 IF CTC assay with respect to HER2 status from patient tumor. (n = number of patients, TP = true positive, FP = false positive, TN = true negative, FN = false negative). (0.05 MB DOC) [file pone.0012517.s009.doc]

|  | ***n*** | ***TP*** | ***FP*** | ***TN*** | ***FN*** | ***Type I error =FP/(FP+TN)*** | ***Type II error =FN/(FN+TP)*** | ***Positive Predicted Value =TP/(TP+FP)*** | ***Negative Predicted Value =TN/(TN+FN)*** |
| --- | --- | --- | --- | --- | --- | --- | --- | --- | --- |
| ***>0 CTC*** | *29* | *9* | *3* | *15* | *2* | *0.17* | *0.18* | *0.75* | *0.88* |
| ***>1 CTC*** | *22* | *5* | *3* | *13* | *1* | *0.19* | *0.17* | *0.62* | *0.93* |
| ***>2 CTCs*** | *18* | *3* | *1* | *13* | *1* | *0.071* | *0.25* | *0.75* | *0.93* |
| ***>3 CTCs*** | *16* | *2* | *1* | *13* | *0* | *0.071* | *0* | *0.67* | *1* |
| ***>4 CTCs*** | *15* | *2* | *1* | *12* | *0* | *0.077* | *0* | *0.67* | *1* |
| ***>5 CTCs*** | *13* | *2* | *1* | *10* | *0* | *0.091* | *0* | *0.67* | *1* |
| ***>6 CTCs*** | *10* | *1* | *0* | *9* | *0* | *0* | *0* | *1* | *1* |
| ***>7 CTCs*** | *9* | *0* | *0* | *9* | *0* | *0* | *NA* | *NA* | *1* |
| ***>8 CTCs*** | *6* | *0* | *0* | *6* | *0* | *0* | *NA* | *NA* | *1* |
| ***<=1 CTC*** | *7* | *4* | *0* | *2* | *1* | *0* | *0.2* | *1* | *0.67* |
| ***<=2 CTCs*** | *11* | *6* | *2* | *2* | *1* | *0.5* | *0.14* | *0.75* | *0.67* |
| ***<=3 CTCs*** | *13* | *7* | *2* | *2* | *2* | *0.5* | *0.22* | *0.78* | *0.5* |
