# Supplementary material for: Molecular Biomarker Analyses Using Circulating Tumor Cells
Source: PLoS One. 2010 Sep 8;5(9):e12517. doi: 10.1371/journal.pone.0012517 (PMC2935889; doi:10.1371/journal.pone.0012517)
Supplement: Supplementary file 3 [file pone.0012517.s001.doc]

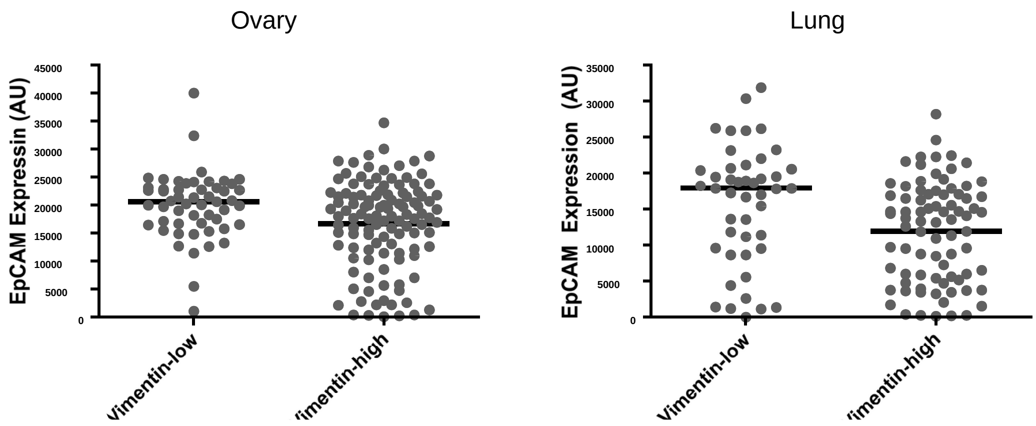

Ovary
Lung
EpCAM Expressin (AU)
EpCAM Expression (AU)
45000
40000
35000
30000
25000
20000
15000
10000
5000
0
Vimentin-low
Vimentin-high
