# Supplementary material for: Molecular Biomarker Analyses Using Circulating Tumor Cells
Source: PLoS One. 2010 Sep 8;5(9):e12517. doi: 10.1371/journal.pone.0012517 (PMC2935889; doi:10.1371/journal.pone.0012517)
Supplement: Supplementary file 4 [file pone.0012517.s002.doc]

a

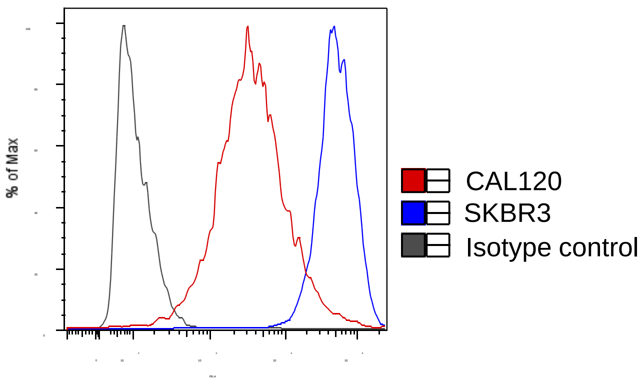
% of Max
CAL120
SKBR3
Isotype control

b

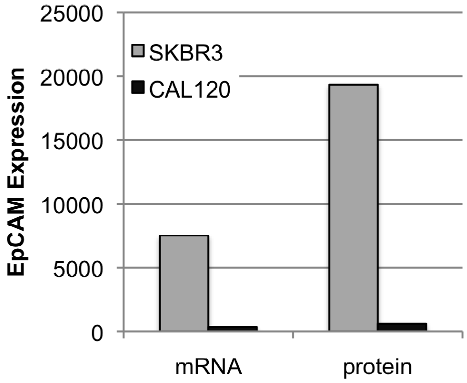
25000
20000
15000
10000
5000
0
EpCAM Expression
SKBR3
CAL120
mRNA
protein
