# Supplementary material for: Molecular Biomarker Analyses Using Circulating Tumor Cells
Source: PLoS One. 2010 Sep 8;5(9):e12517. doi: 10.1371/journal.pone.0012517 (PMC2935889; doi:10.1371/journal.pone.0012517)
Supplement: Supplementary file 5 [file pone.0012517.s003.doc]

The desired number of cells were spiked into donor blood and processed on the different platforms

CTC-Chip

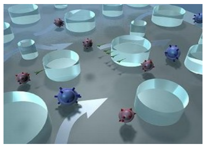

CellSearch

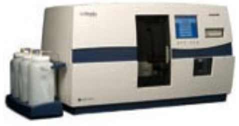

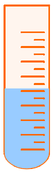

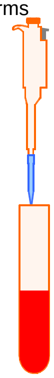

5000cell/ml media suspension prepared
5ul of suspension added to 5 individual slides.
The number of cells counted under the microscope and the mean spiking count determined.

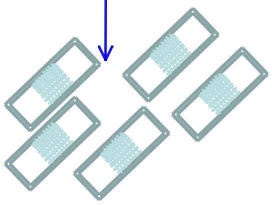

Desired # of cells	Mean spiking count	Cell range	CTC count	CTC recovery rate %
100	78.4	64-88	73	**93**
100	87.2	56-108	80	**92**
100	80.8	72-96	65	**80**
100	82.4	60-100	78	**95**
100	90.4	80-104	81	**90**
100	107.2	80-128	75	**70**
100	93.6	72-120	118	**126**
100	85.6	60-112	78	**91**
100	93.6	76-104	91	**97**
100	104.8	76-124	96	**92**
100	100	80-120	120	**120**
100	100	80-120	116	**116**

+/- 30% error rate.
