# Supplementary material for: Molecular Biomarker Analyses Using Circulating Tumor Cells
Source: PLoS One. 2010 Sep 8;5(9):e12517. doi: 10.1371/journal.pone.0012517 (PMC2935889; doi:10.1371/journal.pone.0012517)
Supplement: Supplementary file 6 [file pone.0012517.s004.doc]

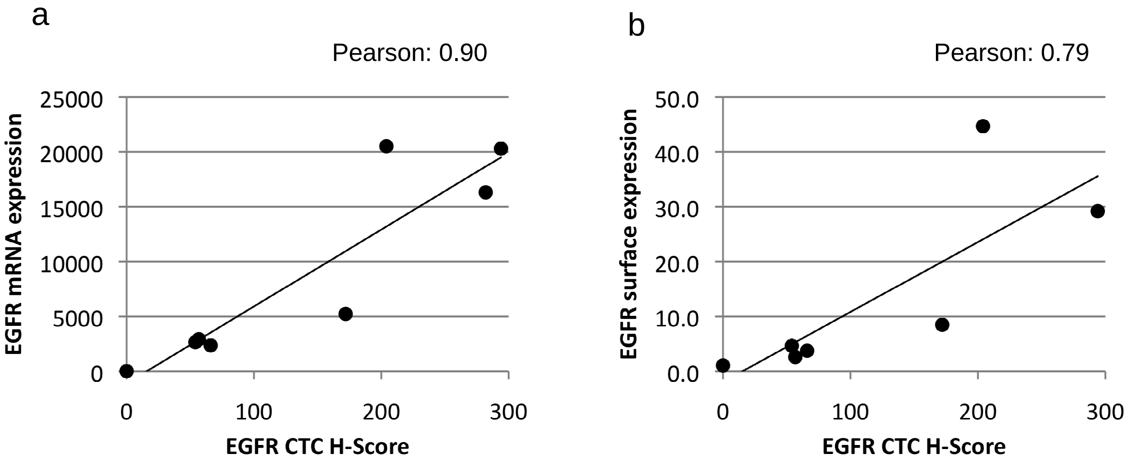
a
Pearson: 0.90
25000
20000
15000
10000
5000
0
EGFR mRNA expression
0
100
200
300
EGFR CTC H-Score
b
Pearson: 0.79
50.0
40.0
30.0
20.0
10.0
0.0
EGFR surface expression
0
100
200
300
EGFR CTC H-Score
