# Supplementary material for: Molecular Biomarker Analyses Using Circulating Tumor Cells
Source: PLoS One. 2010 Sep 8;5(9):e12517. doi: 10.1371/journal.pone.0012517 (PMC2935889; doi:10.1371/journal.pone.0012517)
Supplement: Supplementary file 7 [file pone.0012517.s005.doc]

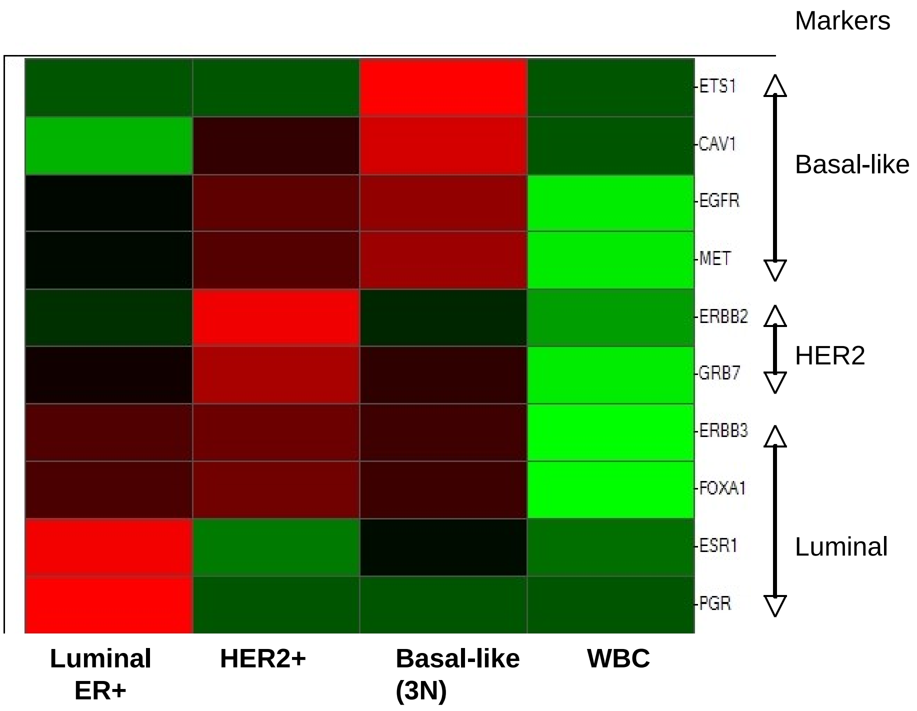

Markers
ETS1
CAV1
EGFR
MET
ERBB2
GRB7
ERBB3
FOXA1
ESR1
PGR
Basal-like
HER2
Luminal
Luminal ER+
HER2+
Basal-like (3N)
WBC
