# Supplementary material for: Molecular Biomarker Analyses Using Circulating Tumor Cells
Source: PLoS One. 2010 Sep 8;5(9):e12517. doi: 10.1371/journal.pone.0012517 (PMC2935889; doi:10.1371/journal.pone.0012517)
Supplement: Supplementary file 8 [file pone.0012517.s007.doc]

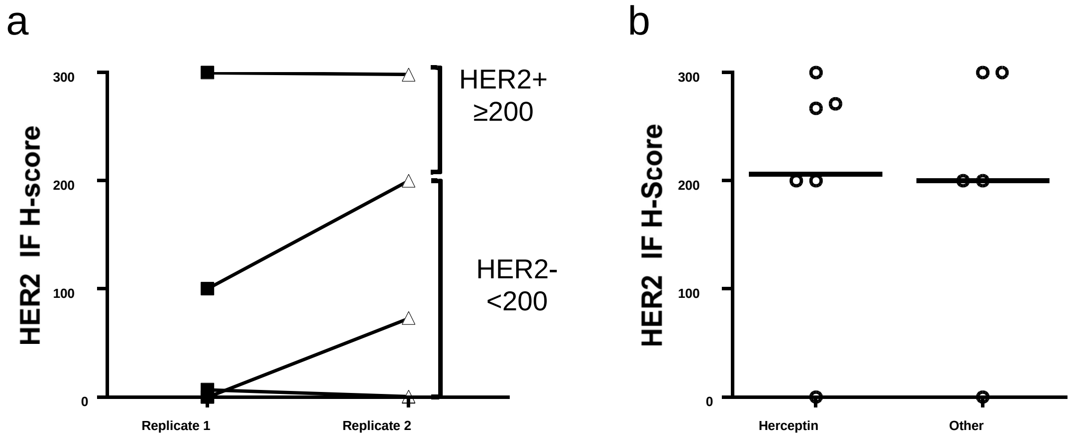
a
HER2 IF H-score
300
200
100
0
Replicate 1
Replicate 2
HER2+
≥200
HER2-
<200
b
HER2 IF H-Score
300
200
100
0
Herceptin
Other
